# An Improved Multidimensional MPA Procedure for Bidirectional Earthquake Excitations

**DOI:** 10.1155/2014/320756

**Published:** 2014-07-20

**Authors:** Feng Wang, Jian-Gang Sun, Ning Zhang

**Affiliations:** ^1^College of Architecture & Civil Engineering, Dalian Nationalities University, Dalian 116600, China; ^2^Dalian Polytechnic University, Dalian 116003, China

## Abstract

Presently, the modal pushover analysis procedure is extended to multidimensional analysis of structures subjected to multidimensional earthquake excitations. an improved multidimensional modal pushover analysis (IMMPA) method is presented in the paper in order to estimate the response demands of structures subjected to bidirectional earthquake excitations, in which the unidirectional earthquake excitation applied on equivalent SDOF system is replaced by the direct superposition of two components earthquake excitations, and independent analysis in each direction is not required and the application of simplified superposition formulas is avoided. The strength reduction factor spectra based on superposition of earthquake excitations are discussed and compared with the traditional strength reduction factor spectra. The step-by-step procedure is proposed to estimate seismic demands of structures. Two examples are implemented to verify the accuracy of the method, and the results of the examples show that (1) the IMMPA method can be used to estimate the responses of structure subjected to bidirectional earthquake excitations. (2) Along with increase of peak of earthquake acceleration, structural response deviation estimated with the IMMPA method may also increase. (3) Along with increase of the number of total floors of structures, structural response deviation estimated with the IMMPA method may also increase.

## 1. Introduction

In recent years, a challenge for performance based seismic design is to establish an effective and feasible procedure to evaluate structural seismic capacities. The pushover analysis procedure, due to its simplicity and efficiency, is increasingly applied to estimate seismic demands of structures. During past years, the theory and application of pushover analysis procedure have been developed adequately [1–7], and the technique is accepted by more guideline documents or codes such as FEMA-440 and Vision 2000.

A problem for pushover analysis is that the higher mode effects of building structures cannot be considered in the procedure [8]. In order to solve this, Chopra and Goel [9–11] propose a modal pushover analysis (MPA) procedure to deal with higher mode effects, in which the seismic demand of individual terms of the modal expansion is determined by pushover analysis using the inertia force distribution for each mode, and the modal demands are then combined by the SRSS rule to obtain the total seismic demand. On the basis of this, some improved approach is presented [12–16]. Another problem is that the pushover procedure is restricted with an assumption of single-mode response in which the deformation distributions along the height of structures are fixed. For solving the problem, adaptive pushover methods [17–23] are presented which aim to capture the changes that occur in the vibration properties of structures.

The previous studies about pushover analysis are almost based on symmetric building structures and single-directional earthquake excitation. Both theoretical studies and seismic disasters indicate that asymmetric-plan structures with irregular distributions of mass or stiffness are likely to undergo torsional responses coupled with the translational vibrations, and this type of structures is likely to suffer more severe displacement demands at the corner elements under earthquake excitations. In addition, the torsion coupling response will induce the structural space effects that cannot be solved in two-dimensional analysis. So, several research efforts have been made to extend and apply the pushover analysis to asymmetric-plan structures whose inelastic seismic responses are intricate [24–26]. At present, the MPA [27–31] is extended to the asymmetric-plan structures in which the inertia force distribution for each mode includes two lateral forces and torque at each floor level, and the CQC rule is used to combine the modal demands to obtain the total demand of asymmetric-plan structures.

For multidimensional MPA, which is applicable to the analysis of asymmetric-plan structures, the modal equivalent single-degree-of-freedom (ESDOF) system is actually subjected to the superposition of bidirectional earthquake excitations. In the current MPA procedures, the response of ESDOF system is calculated according to single-directional earthquake excitation, the *x*- and *y*-axes, respectively, and peak response of two directions is combined through SRSS rule, which is applicable to elastic analysis but not applicable to inelastic analysis for having no regard for inelastic coupling of structure responses caused by earthquake excitations of different components. For solving the problem, an improved multidimensional MPA (IMMPA) idea is presented in the paper: superposed bidirectional earthquake acceleration acting on modal ESDOF system can be regarded as an unidirectional “combined earthquake excitation”; in the procedure, to solve structural responses, seismic demand spectra may be established based on SDOF system subjected to the “combined earthquake excitation.”

The main objective of this paper is to propose an MPA procedure for evaluating the seismic capacities of asymmetric-plan structures subjected to bidirectional earthquake excitations. An outline of this paper can be expressed as follows. First, the improved procedure is presented based on the assumption of modal equivalent SDOF system which is subjected to the superposition of bidirectional earthquake excitations. Then, the corresponding strength reduction factor spectra are discussed and compared with traditional spectra. Next, the step-by-step procedure is proposed. At the end, two examples are considered to verify the accuracy of the proposed procedure.

## 2. Motion Equations of Equivalent Systems

Consider an *n*-storey building. Its plane is asymmetric about the *x*- or/and *y*-axes. Each floor diaphragm is rigid in its own plane and has three degrees of freedom defined at the center of mass (CM). The equation of motion governing the response of the *n*-storey asymmetric structure subjected to bidirectional earthquake excitations (along the *x*- and *y*- axes) is as follows:

(1) $$\begin{array}{c} M\ddot{u}\left(t\right)+C\dot{u}\left(t\right)+F\left(u\right)=-M\iota_{x}{\ddot{u}}_{gx}\left(t\right)-M\iota_{y}{\ddot{u}}_{gy}\left(t\right), \end{array}$$

where **M** is diagonal mass matrix of order 3*n* which includes three submatrices **m**, **m**, and **I**_*o*_; **m** is associated with *x* and *y*-lateral degrees of freedom, and **I**_*o*_ is associated with torsion degrees of freedom. **C** is the damping matrix and **F**(**u**) is the vector of resisting forces. The displacement vector **u**(*t*) is equal to [**u**_*x*_ **u**_*y*_ **u**_*θ*_]^*T*^; **u**_*x*_, **u**_*y*_, and **u**_*θ*_ are the *x*, *y*, and torsion-directional displacement subvectors, respectively. The influence vectors, **ι**_*x*_ and **ι**_*y*_, are as follows:

(2) $$\begin{array}{c} \iota_{x}=\left[\begin{array}{ccc} \left\{1\right\} & \left\{0\right\} & \left\{0\right\} \end{array}\right],\;\;\iota_{y}=\left[\begin{array}{ccc} \left\{0\right\} & \left\{1\right\} & \left\{0\right\} \end{array}\right]. \end{array}$$

In MPA procedure, a major assumption is that the responses of structures can be expressed as superposition of the responses of appropriate SDOF systems just like that in the linear range. The displacement vector of the inelastic system can be expanded in terms of the natural vibration modes of the corresponding linear system:

(3) $$\begin{array}{c} u\left(t\right)=\Phi q=\overset{3n}{\underset{i=1}{\sum }}\varphi_{i}q_{i}\left(t\right), \end{array}$$

in which the *i*th modal vector **φ**_*i*_ of size 3*n* × 1 includes three *n* × 1 subvectors **φ**_*xi*_,  **φ**_*yi*_, and **φ**_*θi*_. Substituting **u**(*t*) = Φ**q** into (1) and premultiplying both sides of (1) by **φ**_*i*_^*T*^, the following equations are given:

(4) φiTMφiq¨i(t)+φiTCφiq˙i(t)+Fsi(t) =−φiTMφi(Γxiu¨gx(t)+Γyiu¨gy(t)), i=1,…,3n,

where the *i*th modal resisting force quantity, *F*_*si*_(*t*), equals **φ**_*i*_^*T*^ **K**_ep_(*t*)**φ**_*i*_ *q*_*i*_(*t*); **K**_ep_(*t*) is elastic-plastic instantaneous stiffness matrices; **φ**_*i*_^*T*^ **M** **φ**_*i*_ is interpreted as the *i*th modal equivalent mass, *M*_*i*_; Modal participation factors, Γ_*xn*_ and Γ_*yn*_, along the *x*- and *y*-axes, respectively, are expressed as

(5) $$\begin{array}{c} \Gamma_{xi}=\frac{\varphi_{i}^{T}M\iota_{x}}{\varphi_{i}^{T}M\varphi_{i}},\;\Gamma_{yi}=\frac{\varphi_{i}^{T}M\iota_{y}}{\varphi_{i}^{T}M\varphi_{i}},\;i=1,\ldots ,3n. \end{array}$$

Dividing both sides of (4) by modal equivalent mass, *M*_*i*_, and using the orthogonality property of modes, 3*n* uncoupled equations can be derived as

(6) q¨i(t)+2ξωiq˙i(t)+Fsi(t)Mi=−Γx,iu¨gx(t)−Γy,iu¨gy(t),i=1,…,3n,

which are the motion equations of equivalent systems from 1th to 3*n*th mode of the considered structure. In (6), *ω*_*i*_ is natural vibration frequency of the *i*th modal equivalent system of the structure and *ξ* is damping ratio. The relationship between the resisting force parameter, *F*_*si*_/*M*_*si*_, and the modal coordinate, *q*_*i*_, of the equivalent systems can be estimated from an idealized base shear and roof displacement pushover curve of the *n*th mode. The equivalent displacement responses, *q*_1_(*t*) ⋯ *q*_*i*_(*t*) ⋯ *q*_3*n*_(*t*), can be calculated by (6), and then the displacement vector, **u**(*t*), can be solved by (3). Transposing $M\ddot{u}(t)$ from the left side of (1) to the right side, the following is given:

(7) $$\begin{array}{c} C\dot{u}+F\left(u\right)=-M\left(\ddot{u}\left(t\right)+\iota_{x}{\ddot{u}}_{gx}\left(t\right)+\iota_{y}{\ddot{u}}_{gy}\left(t\right)\right)=P_{\text{ine}} \end{array}$$

in which **P**_ine_ can be interpreted as earthquake inertia force vector of the structure. Substituting (3) into (7), **P**_ine_ can be expressed as a summation:

(8) Pine=−∑i=13npine,i=−∑i=13nMφi(q¨i(t)+Γx,iu¨gx(t)+Γy,iu¨gy(t))

in which **p**_ine,*i*_ is the *i*th modal inertia force vector of the structure.

## 3. Traditional Multidimensional MPA

In the traditional multidimensional MPA procedure, the peak modal responses *r*_*x*_ and *r*_*y*_ to *x*- and *y*-components of earthquake motion are determined by nonlinear static analysis, respectively. Associated with components of earthquake motion, the total inertia force vector can be expanded as a summation:

(9) Pine=−∑i=13npine,x,i−∑i=13npine,y,i=−∑i=13nMφi(q¨x,i(t)+Γx,iu¨gx(t))−∑i=13nMφi(q¨y,i(t)+Γy,iu¨gy(t)).

In the procedure, the contribution of responses for one component (*x* or *y*) of ground motion is assumed to be uncoupled, and the uncoupled equation of motion is expressed as

(10) $$\begin{array}{c} {\ddot{q}}_{x,i}\left(t\right)+2\xi \omega_{i}{\dot{q}}_{x,i}\left(t\right)+\frac{F_{sx,i}\left(t\right)}{M_{i}}=-\Gamma_{x,i}{\ddot{u}}_{gx}\left(t\right)⟹r_{x,i},\\ {\ddot{q}}_{y,i}\left(t\right)+2\xi \omega_{i}{\dot{q}}_{y,i}\left(t\right)+\frac{F_{sy,i}\left(t\right)}{M_{i}}=-\Gamma_{y,i}{\ddot{u}}_{gy}\left(t\right)⟹r_{y,i}. \end{array}$$

The peak responses of *x*- and *y*-directions are determined, respectively, by combining the peak modal responses using the CQC rule as follows:

(11) $$\begin{array}{c} r_{x}={\left(\underset{i}{\sum }\underset{j}{\sum }\;\rho_{ij}r_{x,i}r_{x,j}\right)}^{1/2};\;\;r_{y}={\left(\underset{i}{\sum }\underset{j}{\sum }\;\rho_{ij}r_{y,i}r_{y,j}\right)}^{1/2}. \end{array}$$

The responses *r*_*x*_ and *r*_*y*_ are combined to determine the total response *r* by the SRSS rule:

(12) $$\begin{array}{c} r=\sqrt{r_{x}^{2}+r_{y}^{2}}. \end{array}$$

In the procedure, the responses of different horizontal components of earthquake excitation are uncoupled. The superposition rule is utilized here to solve the problem which is accurate in linear response stage of the structures under bidirectional ground motions but it is not applicable in nonlinear stage.

## 4. Improved Multidimensional MPA (IMMPA)

### 4.1. Equivalent SDOF System under Superposition Excitation

If abs |Γ_*x*,*i*_|⩾abs |Γ_*y*,*i*_|, extract Γ_*x*,*i*_ from the right side of equation and define Γ_*x*,*i*_ = Γ_*i*_. The motion equation of equivalent systems is then transformed as

(13) q¨i(t)+2ξωiq˙i(t)+Fsi(t)Mi=−Γi(u¨gx(t)+γiu¨gy(t)),i=1,…,3n .

In addition, if abs |Γ_*y*,*i*_|⩾abs |Γ_*x*,*i*_|, a similar equation can be established, and it is omitted here because of theoretical consistency. In (13), the parameter *γ*_*i*_ equals Γ_*y*,*i*_/Γ_*x*,*i*_ which is in the range from −1 to 1. The superposition of bicomponent ground acceleration time history, ${\ddot{u}}_{gx}(t)+\gamma_{i}{\ddot{u}}_{gy}(t)$, in the right side of (13) can be interpreted as a single-component acceleration time history, ${\ddot{U}}_{gi}(t)$, and the corresponding motion equations are reduced to

(14) $$\begin{array}{c} {\ddot{q}}_{i}\left(t\right)+2\xi \omega_{i}{\dot{q}}_{i}\left(t\right)+\frac{F_{si}\left(t\right)}{M_{i}}=-\Gamma_{i}{\ddot{U}}_{gi}\left(t\right),\;i=1,\ldots ,3n. \end{array}$$

Defining *d*_*i*_(*t*) = *q*_*i*_(*t*)/Γ_*i*_, (14) is transformed as

(15) $$\begin{array}{c} {\ddot{d}}_{i}\left(t\right)+2\xi \omega_{i}{\dot{d}}_{i}\left(t\right)+f_{i}\left(t\right)=-{\ddot{U}}_{gi}\left(t\right),\;i=1,\ldots ,3n, \end{array}$$

where *d*_*i*_(*t*) is governed by the equation of motion for the *i*th modal equivalent SDOF system subjected to earthquake excitation, ${\ddot{U}}_{gi}(t)$. The resisting force term, *f*_*i*_(*t*), equals *F*_*si*_(*t*)/*M*_*i*_Γ_*i*_. Equation (15) shows that, due to the aforementioned assumptions, the nonlinear response of a structure system with 3*n* degrees of freedom subjected to bidirectional earthquake excitations can be expressed as the sum of the responses of 3*n* equivalent SDOF systems under single-directional excitation, ${\ddot{u}}_{gx}(t)+\gamma_{i}{\ddot{u}}_{gy}(t)$, each one corresponding to a vibration “mode.”

According to the derivation above, the expression of inertia force vector, **P**_ine_, can be expanded as

(16) $$\begin{array}{c} P_{\text{ine}}=-\overset{3n}{\underset{i=1}{\sum }}p_{\text{ine},i}=-\overset{3n}{\underset{i=1}{\sum }}\Gamma_{i}M\varphi_{i}\left({\ddot{d}}_{i}\left(t\right)+{\ddot{U}}_{g}\left(t\right)\right), \end{array}$$

where the modal inertia force vector, **p**_ine,*i*_, can be reduced as

(17) $$\begin{array}{c} p_{\text{ine}}=s_{i}A \end{array}$$

in which **s**_*i*_ is distribution factor vector of inertia force, and **s**_*i*_ equals

(18) $$\begin{array}{c} s_{i}=\Gamma_{i}{\left[\begin{array}{ccc} m\varphi_{xi} & m\varphi_{yi} & I_{o}\varphi_{\theta i} \end{array}\right]}^{T}. \end{array}$$

The factor vector, **s**_*i*_, can be interpreted as the distribution factor of modal pushover analysis.

### 4.2. Inelastic Response Demands for Equivalent Systems under Superposition Excitation

Generally, the response demands of inelastic systems can be obtained by inelastic response spectra, such as strength reduction factor spectra (i.e., *R*-*μ*-*T* relation [32]).

The yield resisting force expression *f*_*i*,*y*_ = *k*_*x*_ *d*_*i*,*y*_ is defined in which *k*_*x*_ is linear stiffness and *d*_*i*,*y*_ is yield displacement, and the relationship for *μ*_*i*_(*t*) = *d*_*i*_(*t*)/*d*_*y*_ is defined, and (15) can be then normalized as

(19) μ¨i(t)+2ξωiμ˙i(t)+ωi2fi(t)fi,y=−ωi2·Rbiβ(ωi,ξ)U¨g(t)max⁡⁡(|U¨g(t)|),i=1,…,3n,

where *β*(*ω*_*i*_, *ξ*) is amplification coefficient spectrum corresponding to fixed damping ratio, *ξ*, natural vibration frequency, *ω*_*i*_, and excitation; *R*_*bi*_ is strength reduction factor; *R*_*bi*_ equals *f*_*e*,*i*_(*t*)/*f*_*i*,*y*_(*t*) in which *f*_*e*,*i*_(*t*) is elastic resisting force of corresponding elastic equivalent system. By solving (19), the maximal value of *μ*_*i*_(*t*) is ductility factor, *μ*_*i*_, that is defined as the ratio between maximal displacement, *d*_*i*,max⁡_, and yield displacement, *d*_*y*_. Based on (19), the relationship for *R*_*bi*_-*μ*_*i*_-*T*_*i*_ (*T*_*i*_ = 2*π*/*ω*_*i*_) can be established through iterative calculation.

On the other hand, for a SDOF system, according to the definitions of strength reduction factor, *R*_*b*_, and ductility factor, *μ*, the relationship is given as

(20) $$\begin{array}{c} d_{p,\max}=\omega^{2}\cdot A_{e}\cdot \frac{\mu }{R_{b}}, \end{array}$$

where *A*_*e*_ is elastic maximum acceleration response (or elastic acceleration spectra); *d*_*p*,max⁡_ is inelastic maximum displacement response (or inelastic displacement spectra). Substituting *A*_*e*_ and “*R*_*b*_-*μ*-*T*” model into (19), then the inelastic maximum displacement, *d*_*p*,max⁡_, of the SDOF system can be solved.

**Figure 1:**
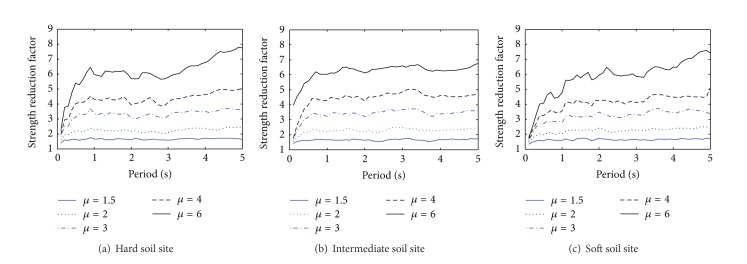
Strength reduction factor *R*_*s*_ spectra.

During the past years, some researchers have presented convenient *R*_*s*_ spectra models [32–34]. To combine with current *R*_*s*_ spectra, it is recommended in this paper to establish *R*_*b*_ spectra by analyzing the relationship between *R*_*s*_ spectra and *R*_*b*_ spectra. The approach is as follows: establish *R*_*b*_/*R*_*s*_ ratio spectra based on different soil sites, analyze the influences of soil site classification, ductility factor, and participation coefficient ratio of vibration mode of two spindle axes, and establish simplified relationship of *R*_*b*_/*R*_*s*_. On this basis, establish *R*_*b*_ spectra with *R*_*s*_ spectra and simplified relation of *R*_*b*_/*R*_*s*_. The *R*_*s*_ spectra for three kinds of soil sites are shown in Figure 1.

**Figure 2:**
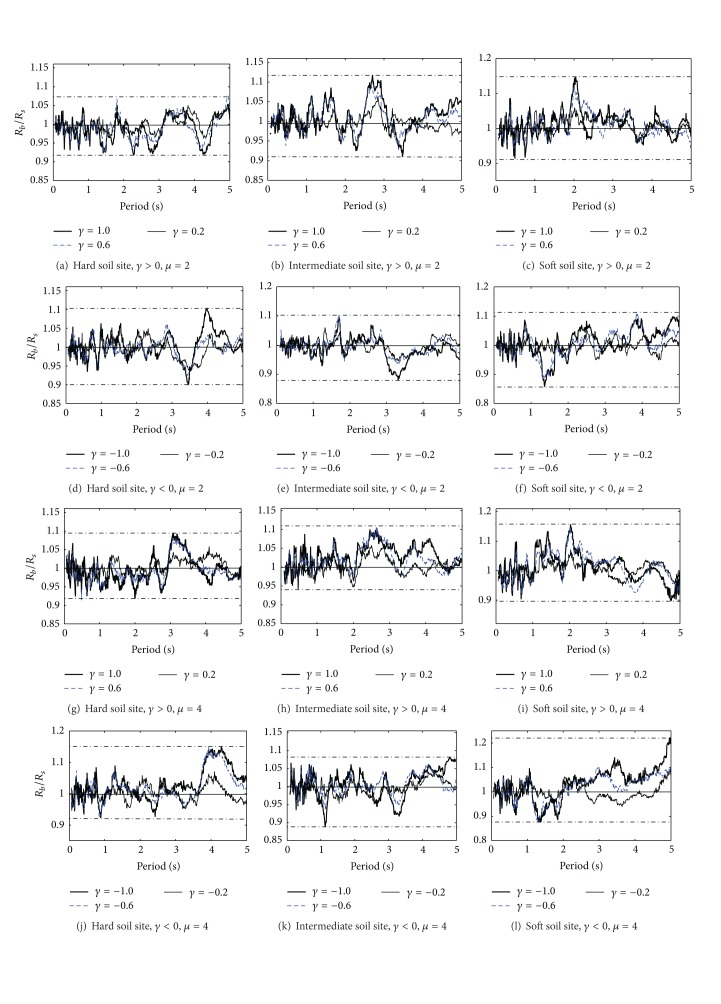
The spectra of *R*_*b*_/*R*_*s*_ for the three kinds of soil sites.

The *R*_*b*_/*R*_*s*_ spectra (*μ* = 2 and 4, three kinds of soil sites) are shown in Figure 2, in which *μ* is ductility factor and *γ* is participation coefficient ratio of vibration mode of two spindle axes. The following can be drawn from *R*_*b*_/*R*_*s*_ spectra analysis. (1) The values of *R*_*b*_/*R*_*s*_ fluctuate around 1 and have no significant regularity as the period increases. (2) The regularity of *R*_*b*_/*R*_*s*_ for *γ* > 0 and that for *γ* < 0 are different from each other, so it is suggested to analyze them, respectively.

**Figure 3:**
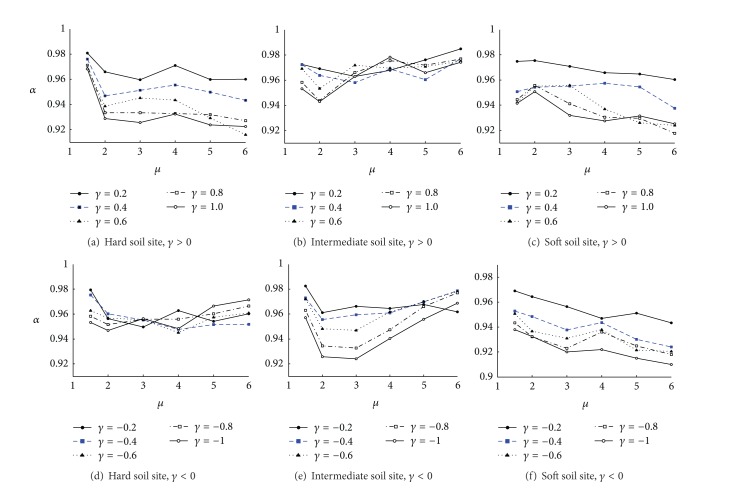
Analysis of modification coefficient *α*.

Through the above analysis, the regularity of *R*_*b*_/*R*_*s*_ along with period change is not very significant, so it is suggested to take the same *R*_*b*_/*R*_*s*_ spectra value within the entire period. In addition, for the reason of disadvantage in *R*_*b*_/*R*_*s*_ < 1, “mean value − 1.65 × standard deviation” of *R*_*b*_/*R*_*s*_ within 0.05~5.0 seconds is defined as modification coefficient, *α*, of traditional *R*_*s*_. The distribution curves of modification coefficient, *α*, along with ductility factor changes are shown in Figure 3. The following can be drawn from Figure 3. (1) For all kinds of soil sites, the minimum value of *α* is 0.910 and the maximum value is 0.985, which shows that the influence of the analysis variables on *R*_*s*_ spectra is little. (2) For *γ* > 0, *α* decreases while *γ* increases; for *γ* < 0, *α* decreases while *γ* decreases (it is not so significant for *γ* < 0 at hard soil site and for *γ* < 0 at intermediate soil site). (3) For hard soil site, *α* is maximized while *μ* = 1.5; *α* decreases and does not change significantly along with increase of ductility factor while *μ*⩾2; for intermediate soil site, *α* increases along with increase of the ductility factor while *μ*⩾2; for soft soil site, *α* decreases along with increase of ductility factor.

### 4.3. Step-by-Step Procedure

A step-by-step summary of the IMMPA procedure is presented as follows.

1. The natural frequencies, *ω*_*i*_, and modes, **φ**_*i*_, for elastic vibration of the multidimensional building are calculated and the corresponding mode shapes are normalized.
2. For the *i*th mode, determine the base shear-roof displacement, *V*_*bi*_-*u*_*ri*_, pushover curve by multidimensional nonlinear static analysis applying the force distribution (17). *V*_*bi*_ and *u*_*ri*_ are chosen to correspond to the direction of the *x*- and *y*-components, respectively. The pushover curve is in the direction of the dominant excitation in the mode being considered.
3. Idealize the *V*_*bi*_-*u*_*ri*_ pushover curve as a bilinear *A*-*D* curve by utilizing the well-known procedure [35].
4. Determine *A*_*y*_-*D* earthquake demand spectra curve based on the nonlinear dynamic analysis of modal equivalent SDOF system subjected to the superposition excitation.
5. Determine the roof object displacement of *i*th mode and then calculate the other *i*th modal responses (such as drifts, plastic rotations, etc.) by conducting pushover analysis up to the already calculated object displacement.
6. Repeat steps 1–5 for as many modes as required for sufficient accuracy.
7. Determine the total extreme responses, *r*, (including *r*_*x*_- and *r*_*y*_-components) for the excitation of the combined earthquake motion by combining gravity response and the peak modal responses by utilizing the CQC rule.

## 5. Numerical Example for IMMPA Procedure

### 5.1. Description of the Example Buildings

**Figure 4:**
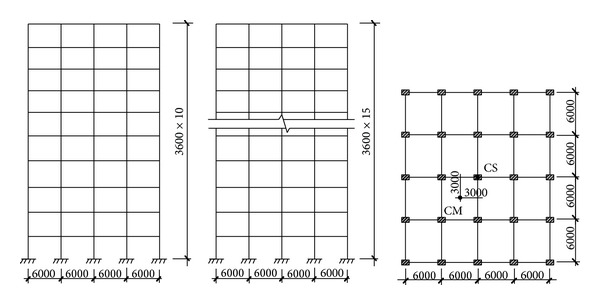
Schematic of example buildings.

In order to clarify how the proposed methodology should be applied, two simple analytical examples are presented. The structures considered are a 10-storey and a 15-storey eccentric reinforced concrete frame buildings, as illustrated in Figure 4. It is considered that the ground motion is acting simultaneously along the two horizontal axes. Each floor has three degrees of freedom (DOF) defined at the center of mass (CM). Each floor diaphragm is rigid in its own plane and the center of stiffness (CS) deviates from the CM. The dimensions of the two buildings in height are 3.6 m for all stories, and the dimensions in plane are shown as Figure 4. For the 10-storey building, the sectional sizes of beams are 300 × 700 mm^2^ for 2th~11th floor; the sectional sizes of columns are 700 × 700 mm^2^ for 1th~3th storey, 600 × 600 mm^2^ for 4th~7th storey, and 500 × 500 mm^2^ for 8th~10th storey. For the 15-storey building, the sectional sizes of beams are 300 × 700 mm^2^ for 2th~5th floor, 300 × 650 mm^2^ for 6th~11th floor, and 300 × 600 mm^2^ for 12th~16th floor; the sectional sizes of columns are 700 × 700 mm^2^ for 1th~4th storey, 650 × 650 mm^2^ for 5th~10th storey, and 500 × 500 mm^2^ for 11th~15th storey. Steel ratios are approximately 1.5% for beam sections and 2% for column sections of the two buildings. Concrete compression strength is selected as 30 MPa for all columns and beams of the structure. The design dead load and live load are, respectively, 6.6 kN/m^2^ (4.7 kN/m^2^) and 1.0 kN/m^2^ (2.0 kN/m^2^) for each floor (roof). The damping of the building is modeled by the Rayleigh damping, and damping ratio *ξ* equals 5%.

**Figure 5:**
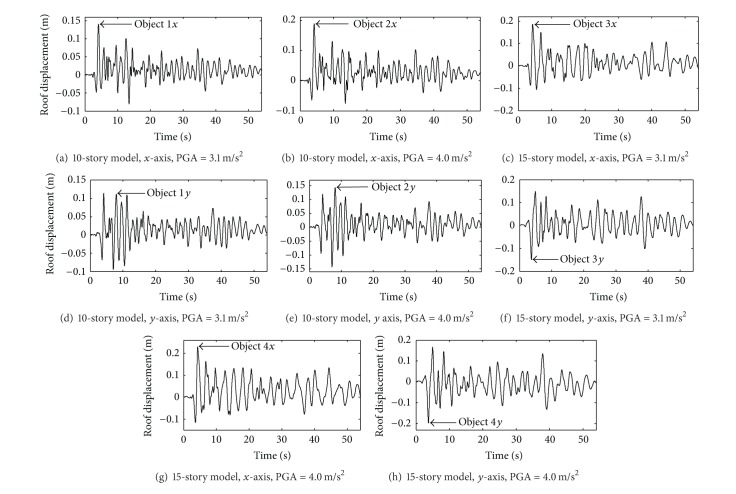
Displacement of roof of the structural models.

The bidirectional “Taft” earthquake records (Kern County 1952, 1095 Taft Lincoln School) are selected to verify the accuracy of the presented method. The acceleration peak values of the records are adjusted to 3.1 m/sec^2^ and 4.0 m/sec^2^, respectively. The nonlinear response time history analyses (NL-RHA) are implemented for the two example building subjected to the selected earthquake record and the time history responses of roof displacements are illustrated in Figure 5, in which 8 peak displacement points are defined as 8 objective control points for variance analysis.

### 5.2. Comparative Evaluation Method

The method to compare estimation results of IMMPA with exact results of NL-RHA for verifying the procedure is as follows.

1. The restoring force model of modal equivalent SDOF system is established by modal pushover analysis.
2. The dynamic response *Q*_*is*_ of the *i*th mode equivalent SDOF system is solved by NL-RHA, in which the restoring force model obtained in step 1 is used. The dynamic response *Q*_*is*_ is converted into the *i*th modal responses *Q*_*i*_, and total structural dynamic responses *Q* can be obtained by superposition of modal responses; that is, *Q* = ∑*Q*_*i*_.
3. Corresponding to the eight objective control points given in Section 5.1, the modal roof objective displacements, *u*_*roi*_, and structural roof objective displacements, *u*_*ro*_, are solved, respectively, according to step 2.
4. The IMMPA is implemented and the modal static responses *Q*_*i*_^*s*^, corresponding to *u*_*roi*_, are obtained. The total static responses, *Q* ^*s*^, are determined by linear combining the response values of each modal pushover analysis; that is, *Q* ^*s*^ = ∑*Q*_*i*_^*s*^.
5. The demand responses are compared between IMMPA and NL-RHA.

### 5.3. Comparison of Responses

**Figure 6:**
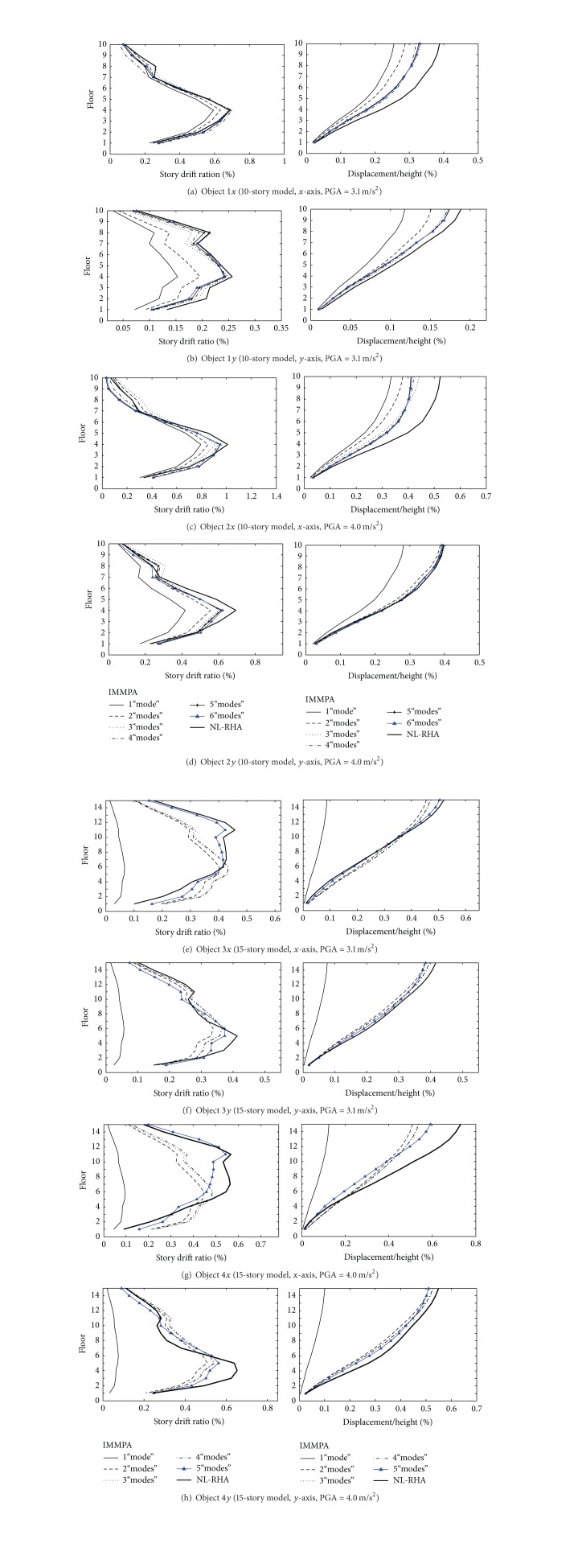
Comparison of deformation responses between IMMPA and NL-RHA.

The comparisons of story drift ratios and floor displacements between NL-RHA method and IMMPA method are illustrated in Figure 6. The values gotten with NL-RHA method are considered to be the exact one, which will be utilized to analyze the estimation accuracy of structural responses with IMMPA method. It can be drawn from Figure 6 that (1) in general IMMPA method can be used to estimate deformation distribution of structures under bidirectional earthquake excitations, rationally. (2) With peak value of earthquake acceleration and structural plastic deformation increase, the deviation of structural response estimated with MMPA may increase. The reason is that the concept of elastic modal decomposition is not applicable to inelastic response analysis in theory. In addition, it is not rational to estimate dynamic response of structures with static analysis method, which may also result in deviation. (3) By comparing 10-storey structure and 15-storey structure in the case, it is found that the response deviation of the 15-storey structure, estimated with IMMPA, is larger than that of the 10-storey structure, so it can be drawn that structural deviation estimated with IMMPA will increase as the number of total floors of structure increases. The reason is that, with the increase of the number of total floors, the vibration modes are gradually complex and the effect of the higher mode on structural response increases gradually.

## 6. Conclusion

A new IMMPA idea is presented in the paper based on traditional MPA method: superposed bidirectional earthquake excitation acting on modal ESDOF system can be regarded as an unidirectional “combined earthquake excitation”; in pushover procedure, the static force replacing “combined earthquake excitation” is assigned to three components of each floor of a structure based on model of vibration.

In accordance with this idea, in solution of structural displacement response, it is required to input “combined earthquake acceleration” to modal ESDOF system in order to establish *R*_*b*_ spectra and convert it to seismic demand spectra. It is recommended in this paper to get modification coefficient of *R*_*s*_ spectra by analyzing the relation of *R*_*b*_/*R*_*s*_. Define modification coefficient, *α*, as “mean value − 1.65 × standard deviation” of *R*_*b*_/*R*_*s*_ within 0.05~5 seconds. Based on selected earthquake records, analyze the influence of soil site classification, ductility factor, and participation coefficient ratio of vibration mode on *α*.

In order to check accuracy of the IMMPA procedure presented in this paper, two structures with mass eccentricity are designed, a bidirectional earthquake ground motion, Taft, is taken as earthquake excitation of the structural system, and deformation distribution of the structure is calculated, respectively, with NL-RHA and IMMPA, and the results are concluded as follows after comparative analysis.

1. In general, IMMPA method can be used to accurately estimate the distribution feature of plastic deformation of the structure along its floors.
2. Along with increase of peak of earthquake acceleration, structural response deviation estimated with IMMPA method may also increase.
3. Along with increase of the number of total floors of structures, structural response deviation estimated with IMMPA method may also increase.
